# New approach for health assessment of high voltage motor using experimental case studies

**DOI:** 10.1371/journal.pone.0342076

**Published:** 2026-02-27

**Authors:** Salem Mgammal Al-Ameri, Moorthy Ramasamy, Waleed M. Hamanah, Mohd Fairouz Yousof, Samir Ahmed Al-Gailani, Ali Ahmed Salem

**Affiliations:** 1 Applied Research Centre for Metrology, Standards, and Testing, Research and Innovation, KFUPM, Dhahran, Saudi Arabia; 2 Department of Electrical Power Engineering, Faculty of Electrical and Electronic Engineering, University Tun Hussein Onn Malaysia, Batu Pahat, Johor, Malaysia; 3 Department of Electrical Engineering, College of Engineering and Physics, KFUPM, Dhahran, Saudi Arabia; 4 Department of Electrical Engineering, Faculty of Engineering, Aden University, Aden, Yemen; Aalto University, FINLAND

## Abstract

This paper introduces a new approach for comprehensive Health Index (HI) assessment of high-voltage (HV) induction motors used in oil and gas plants. The proposed method is designed to be practical, relying on readily available operational and maintenance data without requiring the installation of additional sensors. It accounts for real-world limitations in data acquisition and incorporates internationally recognized criteria from IEC, IEEE, and CIGRE standards. The Health Index calculation integrates both conventional diagnostic test results, such as Partial Discharge (PD), Insulation Resistance (IR), Polarization Index (PI), Tan Delta (TD), vibration measurements, and complementary information/ Conditional Factors CF, including physical condition, maintenance history recorded in the Standard Assessment Procedure (SAP), and aging factors. Condition ratings, weighting factors, and parameter-specific scoring are systematically applied to provide a balanced assessment. By adopting a multi-criteria analysis framework, the proposed method consolidates diverse parameters into a unified, condition-based Health Index. The significance of this work lies in its ability to support proactive asset management, minimize unexpected failures, and ensure the reliable and efficient operation of HV motors. Experimental case studies further demonstrate the applicability and robustness of the approach.

## Introduction

HV motors are electric motors designed to operate at voltage levels greater than 1kV. Common voltage ratings include 3.3kV, 6.6kV, 11kV, and 13.8kV. These motors are commonly used in various industrial applications where high power is required [1]. To achieve the optimal balance among capital investments, asset maintenance costs, and operating performance, there is a need to provide economic and technical justifications for management decisions on capital replacement or job scope identification for major shutdown or maintenance activities [2].

The Health Indices (HI) typically represent a practical method to gauge the field inspections and site testing into a quantitative index, providing the health of the assets [3]. Asset HI has been developed and utilized in many major electrical equipment, such as LV motors, HV Motors, transformers, switchgears, substation, and GIS. The purpose of this approach is to present a condition-based asset management tool that quantifies an HV Motor degradation and allows for an engineering decision-making.

Several studies have examined different HV Motor condition assessment and life-management techniques. These techniques include measuring or monitoring of insulation resistance, partial discharge, thermal imaging, tan delta, vibration monitoring, oil analysis (for motor with a lubrication system), bearing inspection, temperature monitoring, and motor current signature analysis [3]. Such tests are conducted on a routine or condition basis to evaluate the condition of the HV Motor. Other than these tests, some useful data are usually available to evaluate the long-term condition of HV Motor, such as motor age and maintenance data. Although several health indices for HV or LV motor have been developed based on these data, no method is available to quantify the overall condition of the asset by considering indirect aging or deteriorating factors through combining all available data [1]. This paper describes a practical approach to evaluating a comprehensive health index calculation method that combines the motor health index and the conditional factor. It utilizes criteria based on the industry’s common practices, previous studies, and international standards such as IEC, IEEE, and CIGRE recommendations in developing the scoring and ranking methods [2–5].

Even though there are many detection methods on motor failure, online or offline monitoring methods, and health index calculation, there is no specific approach to determine the overall or comprehensive health index. The Health index includes invisible aging factors such as years in operation, the environment of installation, and spare availability. Commonly in industry, engineers only make decisions based on is the CBM results, which leads to an incorrect maintenance strategy for that particular motor, whenever required by management to decide the condition of a motor. They might be uncertain about whether they need to perform minor service, major service, buy critical parts, or replace the asset. No data visualization tools to show the management a summary of the engineering decisions made & prioritize the maintenance or investments, for instance, to purchase spare parts, complete a new unit, or determine the servicing job scope.

This research aims to identify the key parameters that should be considered in developing a Health Index and Conditional Factors (CF) for HV motors. It further seeks to establish a systematic formulation for the Motor Health Index, Conditional Factors, and a Comprehensive Health Index, designed to serve as an engineering decision-support tool for HV motor asset management. In addition, the study applies and evaluates the proposed formulation on real case scenarios involving several HV motors, thereby validating its practicality and effectiveness in industrial environments

### Background on HV induction motor-health index and testing assessment

The HV motor is an electric motor that operates on the principle of electromagnetic induction to convert electrical energy into mechanical energy. It is called “High Voltage” because it typically operates at higher voltage levels, often in industrial or power distribution applications [4]. The stationary part of the motor is called the stator, which contains coils of wire connected to the power supply. When an alternating current (AC) is applied to these coils, it produces a rotating magnetic field. Inside the stator is the rotor, which is a rotating component [5]. The rotor is not connected to any external power source but is influenced by the rotating magnetic field produced by the stator. The changing magnetic field induces an electric current in the rotor conductors according to Faraday’s law of electromagnetic induction. This induced current creates its own magnetic field, and the interaction between the rotor and stator magnetic fields causes the rotor to turn [6]. As a result of this interaction, the rotor starts to rotate, driving the mechanical load connected to the motor shaft. HV motors are often used in high-power applications. The use of high voltage allows for more efficient power transmission and reduced electrical losses. They find widespread use in industries such as manufacturing, oil and gas, and power generation [4].

Failures in HV motors can result from various factors, and they can impact different components of the motor. Here are some common failures associated with HV motors due to factors like operating conditions, environmental influences, or manufacturing issues [7]. HV Motor winding insulation failure in the motor may lead to a short circuit or open circuit of the motor. This usually leads to an overcurrent earth fault or an overcurrent short circuit. Bearing wear could happen due to continuous operation with inadequate or excessive lubrication, and also due to motor misalignments. Overheating of the motor could happen due to excessive loading or an insufficient cooling system. Electrical imbalance is another type of fault that could affect the motor due to voltage fluctuation or harmonics [5].

### Health index methods

Assessing the health of HV motors requires systematic methods that integrate diagnostic test data, operational parameters, and maintenance history into a single metric. The concept of HI has been widely applied to electrical assets such as motors, transformers, and switchgear. This provides a quantifiable measure of asset condition and expected life. Several approaches have been proposed in the literature, ranging from offline diagnostic testing (e.g., insulation resistance, polarization index, tan delta, and partial discharge) to real-time monitoring methods that utilize intelligent sensors, machine learning, and criticality classifications. In most cases, the Health Index is formulated through weighted scoring and normalization of parameters. This allows results to be categorized into condition levels such as Excellent, Fair, Poor, or Very Poor. Each condition is linked to remaining life expectancy and recommended maintenance actions.

Recent studies have also extended Health Index methodologies to include conditional factors CF, process criticality, and advanced data-driven techniques. These approaches not only improve the accuracy of asset assessment but also support proactive maintenance planning, reduce downtime, and enhance system reliability. Within this context, applying and refining Health Index techniques for HV motors is essential. This is for industries’ maintenance process, such as petrochemicals, oil and gas, and power generation, where motor reliability directly impacts productivity and safety. The following subsection will explain all the methods clearly, outlining both traditional diagnostic-based techniques and emerging data-driven approaches for HV motor health index assessment.

The Health Index & Life Expectancy of HV Motor is presented in [3]. This research explores the factors affecting the life expectancy of HV motors, focusing on insulation ageing due to electrical, thermal, and mechanical stresses. This research established the OHI_M_ based on the Partial Discharge Measurement, Tan-delta Test, Step Voltage Test, Insulation Resistance Test, and Dielectric Discharge. The motor condition is categorized into Excellent, Good, Fair, Poor, and Very Poor, each indicating different life expectancies. However, the findings emphasize health index is a vital tool for quantifying the condition of HV motors. Other factors that are not considered, like the environment of installation and other CF.

The real-time motor HI prediction is presented in [1], and the focus was primarily on real-time data collected from intelligent sensors. This research included only the mechanical and electrical parameters, such as vibration, temperature, current, power, and flow rate. The paper uses machine learning models, particularly Particle Swarm Optimization (PSO), to predict the health index. The paper does consider the criticality of the motor in the process as part of the health index calculation. Based on motor classification:

- Class S: Impact on people or the environment.
- Class A: Sudden production disruption and/or off-specification product.
- Class B: Production disruption and/or off-specification product, but not sudden.
- Class C: No direct effect on product specification.

These classifications help prioritize maintenance actions based on the motor’s importance to the production process and potential consequences of failure.

- Class 0: New machine or new installation.
- Class 1: Acceptable for long-term operation.
- Class 2: Unacceptable for long-term operation; requires condition monitoring or mitigation plan.
- Class 3: Immediate corrective action needed; operating conditions must be adjusted or corrective action performed immediately.

These classifications help in determining the appropriate maintenance actions based on the motor’s condition. Environmental conditions, criticality of the motor in the process, and maintenance history are not being considered. In [8], a methodology is introduced to calculate HI for Permanent Magnet Synchronous Motors (PMSM) and converters. This method uses online condition monitoring parameters. For PMSM, parameters include motor voltage, current, vibration, and temperature. For converters, parameters include DC link capacitance, ESR (Equivalent Series Resistance), and thermal resistance. The scores range from 1 to 5, with 1 indicating “very good” condition and 5 indicating “very poor” condition. The Health Index is normalized using a formula that combines scores and weights. The overall System HI is calculated by combining both PMSM and converters’ individual health index, with a weightage of 60% and 40%, respectively. The formula to calculate the overall Health Index is given by equation (1) below.


$$H\cdot I=\sum \left(S_{max}{xw}_{i}\right)\sum \left(S_{i}{xw}_{i}\right)x\text{1}\text{0}\text{0}\%$$ (1)

Where:

- ($S_{i}$) is the score of each assessment condition,
- ($S_{max}$) is the maximum score
- ($W_{i}$) is the weight.

The health condition of the system is defined based on the Overall Health Index (HI) as follows: Good Condition for HI ≤ 0.4, Moderate Condition for 0.4 < HI ≤ 0.7, and Bad Condition for HI > 0.7. By this, it can detect faults, predict the remaining useful life (RUL) of the equipment, enhancing reliability, and preventing potential failures, especially in critical applications like electric aircraft (eVTOL). This research considers the PMSM and online condition monitoring.

Other research proposed a methodology for evaluating the condition of the insulation system of induction motors using a health index. This index combines expert criteria with real data from repaired motors to guide decision-making for motor maintenance [9]. The offline CBM method applied, mainly Dielectric Tests such as insulation resistance test, polarization index, and dielectric absorption ratio. Weight scoring method and expert’s criteria considered in developing H.I. Real data obtained and H. I calculated the imposed to validate the theoretical values. The outcome HI is presented as “Very Bad” to “Excellent. However, the method can be applied to both HV & LV motors; the method of conducting a dielectric test is the same, just varying in test voltage level only. The Health Index formula is given by equation (2) below.


$$H\cdot I=i=\text{1}\sum n\left(N_{i}{xw}_{i}\right)$$ (2)

Where:

- ($N_{i}$) is the normalized value of the (i)-th test.
- (n) is the total number of tests.
- ($W_{i}$) is the weight assigned to the (i)-th test.

In [10], a research paper focused on HV motors in the Petrochemical Industry. Although this paper does not mention the health index calculation, the data from this study are valuable for our consideration. The failure rate was measured based on multiple parameters. For example the age, operating conditions, average running hours, maintenance interval, and fault occurrence. Common faults such as bearing faults, stator winding faults, and rotor faults were discussed.

Other research in [4,6,11,12] calculates the OHI, but this was on the power transformer, switchgear, and circuit breaker equipment. The calculation involves condition ratings, weighting factors, and assigned scores for specific condition parameters. Employing a multi-criteria analysis approach, the method integrates diverse factors to establish a comprehensive, condition-based Health Index for power transformers. The methodology-based health assessment for the power transformer and HV substation system was proposed in [13,14]. A weighted scoring approach is used to calculate the health index. This weighted average score is then adjusted based on visual inspection, maintenance completeness, and equipment failure rate. Finally, the health index for 15 substations was determined.

### Testing assessment technique

There is a range of diagnostic and monitoring techniques applied to evaluate both electrical and mechanical integrity. These testing methods provide valuable insights into insulation performance, winding condition, thermal behaviour, and overall operational health, forming the foundation for preventive maintenance and comprehensive health assessment.

The Insulation Resistance (IR) test is a type of electrical test performed to evaluate the integrity of insulation in electrical equipment and systems. The primary purpose of this test is to ensure that the insulation material between conductive components is effective in preventing leakage current and maintaining the isolation of different electrical circuits [15]. During the IR test, a high-voltage DC is applied between the conductors or between the conductors and the ground. The winding rated and the applied HV DC are shown in Table 1. The measured resistance indicates the quality of the insulation. Table 2 shows the minimum IR values with the description according to IEEE std. The test helps identify potential issues such as moisture ingress, contamination, or deterioration of insulation materials. The minimum IR value depends upon the rated line voltage of the equipment. Minimum IR as per best practice in a petrochemical plant here is 2(kV + 1) MΩ [16]. Thus, for 6 kV Equipment, the minimum IR value between the line and ground will be = 14 MΩ. There are conductive particles such as the free water sprays on the surface of insulation, carbon dust, surface humidity, metal dust, and some basics from a fault, which can lead to the charging current directions and to be steady. In the case of an index of P.I. = 1.0, the current will appear at 1 minute’s time; then the issue caused by the condition will not be presented, and then it needs action. In the case of P.I. is more than >7, the temperature will rise, and the insulation will overheat. This can be overcome by cleaning and drying the conduction current, and then the charging current will be at a steady state for a longer time (more than 1 minute). In this regard, P.I. does not consider additional measurement but is the insulation resistance at a longer time taken for the measurement [17,18].

**Table 1:** The DC applied during the insulation resistance IR testing (Guidelines from IEEE Std. 43).

Winding rated voltage	DC test voltage during the Insulation Resistance test
< 1,000	500
1,000-2,500	500−1,000
2,501−5,000	1,000-2,500
5,001-12,000	2,500−5,000
> 12,000	5,000-10,000

**Table 2:** From the IEEE Std. 43, the overall minimum IR values in MΩ at 40 °C.

Minimum insulation resistance MΩ	Test specimen
IR_1_ min = kV + 1 IR_1_ min = 100	For machine windings manufactured before 1970 For 1970 and after the most common AC machine windings (form-wound coils)
Notes: • IR1 min: At 40 °C, the minimum value of IR of the entire machine winding • kV: The rated voltage of the machine (terminal-to-terminal voltage, in rms kV) • Insulation evaluation can be used by historical IR1 min values. This is for machines that can not obtain the insulation values from the surface area. • Table 2, in some cases, may not be applicable, for instance, overhang is treated with some grading material	

Polarization Index (PI) is a measure used in insulation resistance testing to assess the condition of insulation in electrical equipment. It is the ratio of the insulation resistance measured at a longer duration to the insulation resistance measured at a shorter duration. The PI minimum values for thermal classes are shown in Table 3. The PI is often expressed as a dimensionless value and is calculated using the following equation (3).

**Table 3:** The recommended P.I value based on the IEEE standard [19].

Thermal Level (Class Rating)	P.I (Minimum value)
A	1.5
B	2.0
H	2.0
F	2.0


$$PI=\frac{R\text{1}\text{0}}{R\text{1}}$$ (3)

where:

- *R*10 is the insulation resistance measured at 10 minutes, and
- *R*1 is the insulation resistance measured at 1 minute.
- Typically, a polarization index greater than 2 is considered acceptable, indicating a healthy insulation system.

Industry acceptance criteria recommend that the winding resistance between phases should be within 2% of each other. Additionally, comparison may also be made with original factory-measured data, where differences within 5% are considered satisfactory [20].

**Fig 1:**
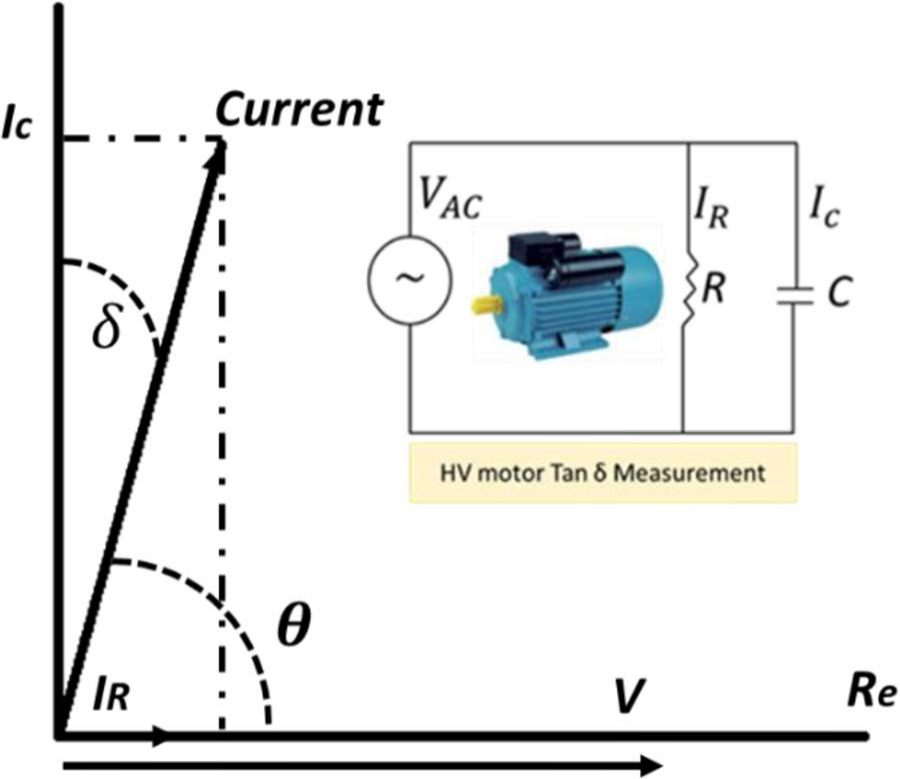
Currents during A. C. voltage supply and dissipation factor (Tan Delta) angle.


The Tan Delta TD test, also known as the power factor test or dissipation factor test, is a diagnostic test conducted on electrical insulation systems to assess their condition. This test is commonly performed on high-voltage equipment, such as transformers, cables, and bushings, to identify potential insulation issues [15]. TD, also known as dielectric dissipation factor. It measures the dielectric losses in the winding insulation as shown in Fig 1. The tan delta is the phase angle between the resistive and capacitive current as given in equation (4).

$$tan\;\delta =\frac{I_{r}}{I_{c}}$$ (4)

Measurement of dielectric dissipation factor is an appropriate method for measuring the quality of new and also aged stator winding insulation of a rotating machine. The method is useful for assessing the uniform quality of manufacturing and the dielectric behaviour of the insulation as a whole. For aged stator windings, the dielectric dissipation factor provides information about insulation condition [21]. The dielectric dissipation factor measurements do not indicate the insulation quality. It is the opposite of the PD offline, which can locate the insulation weak points in the machine. This is the main principle in measuring the dissipation factor [2]. The empirical limits established in practice can be used as a basis for assessing the quality of the winding insulation system during manufacturing. Also, trend assessments (e.g., the functional assessment of the insulation system or diagnostic tests associated with the repair and overhaul of rotating motors) can provide information on the aging process, the need for further measures, and the overhaul intervals. However, they cannot be used to predict the time to failure of the stator winding insulation [22]. As per IEC 60034-27-3:2015, the maximum allowable initial tan delta, delta tan delta, and tan delta tip up of new single bars and coils can be seen in Table 4.

**Table 4:** Dielectric dissipation factor (maximum value) of single bars and coils in new condition with guard ring electrodes up to a rated voltage of Un = 21 kV [22].

NO	Characteristic values measured at room temperature	
1	TD at 0.2Un, (TD 0.2) (the initial value)	20 × 10−3 (2.0%)
2	Per 0.2Un up to Un, (Δ TD/ 0.2Un) (Delta tan delta)	5 × 10−3 (0.5%)
3	Between 0.6Un and 0.2Un, (TD 0.6 – TD 0.2) (Tan delta tip-up)	5 × 10−3 (0.5%)

**Fig 2:**
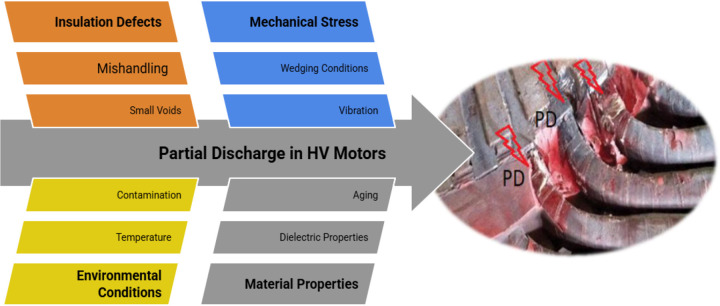
Sources of partial discharge in a stator winding insulation, as in (EC&M Oct. 18, 2018).

Partial Discharge (PD) testing is a diagnostic method used to assess the condition of insulation in high-voltage electrical equipment, including HV motors. PD refers to localized breakdowns or discharges in a small portion of the insulation. The test helps identify potential insulation defects or weaknesses that may lead to electrical failure if left unaddressed. The PD test is designed to be applied to the machines or insulators where the dielectric properties of the insulation material are not in the same condition. This insulator is exposed to partial breakdown or insulation failure [23]. This process may result in several PD during one cycle of the applied voltage. In rotating machines like motors with mica insulation, the occurrence of numerous imperfections like small voids or other PD causes in the new insulation and delamination at aged windings is unavoidable, as shown in Fig 2. Therefore, a superposition of PD sources of different intensities will always be measured [10,23]. Stator winding insulation systems, including type II machines (form-wound coils used in medium-to-high voltage machines) as defined in IEC 60034-18-42, are expected to experience PD activity in service. The time to failure may be independent of the level of partial discharge, but rather depends significantly on many factors, such as the limited operating temperature, wedging conditions, and the degree of contamination [10,23].

Motor Current Signature Analysis (MCSA) is a diagnostic technique used to assess the health and condition of electric motors by analyzing the current waveform during operation it involves monitoring and analyzing the electrical current flowing into the motor to identify specific patterns or signatures associated with various mechanical and electrical issues [9]. It detects the rotor bar defects, where broken or damaged rotor bars can result in characteristic sidebands around the fundamental frequency. Besides, bearing faults can be detected from high-frequency impulses in the current waveform. Misalignment issues can be detected as well as the result in higher harmonics in the current spectrum [9,24,25]. The overall analysis for motor HI testing is discussed in Table 5.

**Table 5:** Review summary on HV Motor Testing Technique.

Reference	Testing Technique	Method of health assessment	Limitations
[15–18]	Insulation Resistance (IR) Test	IR_1_ min = (kV + 1) MΩ IR_1_ min = 100 MΩ	The minimum IR represents the winding IR’s best value before and after 1970. The values may not be applicable in some cases
[19]	Polarization Index (P.I) Test	$P.I=\frac{R\text{1}\text{0}}{R\text{1}}\;$ P.I > 2 acceptable value R10 is the insulation resistance measured at 10 minutes *R*1 is the insulation resistance measured at 1 minute	Typically, a polarization index greater than 2 is considered acceptable. The test applies a DC voltage, which may not replicate the actual stress of AC that occurs during the operation.
[20]	Winding Resistance Test	Winding resistance between phases should be within 2% of each other according to IEEE std. 43	This test measures the winding resistance and cannot evaluate the overall condition of the HV motor
[2], [22–24]	Tan Delta Test TD	As per IEC 60034-27-3:2015, Initial value of TD < 0.5% Delta tan delta ≤ 0.5% Tan delta tip-up ≤ 0.1%/kV	Tan Delta can be used to detect the insulation degradation and ageing, but it should align with other tests, like partial discharge, to estimate the overall condition of the HV motor.
[10], [10,23]	Partial Discharge Test PD	According to IEC 60034-18-42, the PD inception voltage ≥ 1.5 times phase to ground voltage and at any test point ≤ 100 pC – 250 pC).	The test is not a standalone test and needs to be used together with other tests, like the Tan Delta and insulation resistance tests.
[9], [24,25]	Motor Current Signature Analysis MCSA	This is a comparable test with the baseline data	This needs baseline data. MCSA is a normally dependent test.

### Designed health index (H.I) assessment methodology

According to [26], the types of tests, acceptance criteria, and weightage for HV Motor Health Index are proposed. This proposal is amended based on the current practice of the targeted plant. To improve the accuracy of the assessment, invisible ageing factors such as age, actual operating condition, operating ambient conditions, satisfactory in operation, and historical failure record are introduced as a Conditional Factor (CF), which is then used to adjust the overall health index (OHI) percentage of the HV Motor. Based on the OHI, the life expectancy of the machine will be proposed. As the health index is not directly related to life expectancy, it can give an idea of life expectancy by considering some parameter in constant [27].

### Input parameters for HV motor health index

The input parameters to be considered and the weightage are defined based on the previous study done in [3,6,9,12,28–30], expert elections, and the sensitivity and decision impact. For example, in [3,9], the authors emphasize that the IR is the main contributor to the motor HI assessment. Therefore, this received the highest percentage.

Also, an expert election was used for the weightage percentage adopted for both testing and CF on motor HI assessment. As some of the electrical testing, such as Insulation Resistance (I.R), Polarization Index (P.I), Tan Delta (TD), Partial Discharge (PD), they judged as high percentage because they directly reflect the insulation ageing, and electrical stress. The vibration analysis was relatively lower weightage as it reflects the mechanical condition, and it is an external factor.

In addition to expert knowledge and previous studies, the qualitative sensitivity analysis was considered. During the industrial case studies, the changes in the high-weightage parameters, such as years under operation, indicate significant shifts in the OHIM. But variation in the low-weightage parameter, like the spare part, shows a secondary effect on the OHIM.

For the CF, the years under operation, the environment of insulation, and the number of major breakdowns were assigned high weightage due to considering the long-term influence on the insulation ageing. The combination of the three considerations, literature, expertise, and qualitative analysis during the testing, ensures the reasons for choosing the weightage percentages.

Therefore, the weighting method is based on the industry common practices and recommendations from international standards such as the International Electrotechnical Commission (IEC), the Institute of Electrical and Electronics Engineers (IEEE), and CIGRE.

- IEEE Std 43: Used to define guidelines for DC test voltages and minimum acceptable values for Insulation Resistance (IR) and Polarization Index (PI).
- IEC 60034-27-3:2015: Provides the basis for Tan Delta limits, specifically the maximum allowable initial values for new and aged stator winding insulation.
- IEC 60034-18-42: Referenced regarding Partial Discharge (PD) activity and its role as a symptom of insulation deterioration.

The motor health index weightage is as follows:

- Insulation Resistance (30%): Given the highest weight because it is a primary indicator of conduction problems and insulation quality.
- Polarization Index (20%), Tan Delta (20%), and Partial Discharge (20%): These diagnostic tests provide critical data on dielectric behavior and insulation weaknesses.
- Vibration Analysis (10%): Used to detect mechanical issues such as bearing faults or misalignment.

For the conditional factor it is as follow:

- Years in Operation (30%): Justified by studies showing that failure rates increase significantly after 11–25 years of operation.
- Environment of Installation (20%): Based on research indicating that motors near seawater or in corrosive environments have failure rates 4–7 times higher than those in general environments.
- Major Breakdowns (20%): Reflects the impact of historical failure data on current asset health.
- Physical Condition (10%), Criticality (10%), and Spare Availability (10%): These provide additional context for engineering decisions regarding maintenance or replacement.

The most easily available data proposed to be used as diagnostic methods are insulation resistance, polarization index, Tan Delta, Partial Discharge, and Vibration analysis. The scoring method is used for categorizing the condition into several levels. In this case, the score is assigned from 1–3 and 5, with score 1 being the lowest score (bad) and 5 being the highest score (good). A similar concept is available in literature for the transformer Health Index calculation using the Dissolved Gas Analysis, Oil Insulation, and Furan parameters [11]. The parameters, weightage for respective parameters and scoring, and their description are being extracted and tabulated in Table 6 below.

**Table 6:** Parameters, weightage for respective parameters, scoring, and their description.

No.	Type of Test	Proposed Weightage%	Acceptance Range	Scoring Description		
				1	3	5
1	Insulation Resistance (I.R)	30	2(kV + 1) MΩ	< 2 (kV + 1) MΩ	2(kV + 1) MΩ – 1GΩ	>1 GΩ
2	Polarization Index (P.I)	20	> 2	< 2	2	> 3
3	Tan Delta (TD)	20	< 2%	> 10%	10% − 2%	< 2%
4	Partial Discharge (PD)	20	20000	≥ 20000	15000	≤ 5000
5	Vibration Analysis (VA)	10	Danger	Danger	Alert	Good

Based on selected parameters or inputs, the calculation will be obtained. The Percentage of HV Motor Health Index (%HI_M_) is calculated by using equation (5) below.


$${\%HI}_{M\;}=\frac{\sum_{i=\text{1}}^{Q}\left(S_{M;i\;}\times \;W_{M;i\;}\right)}{\sum_{i=\text{1}}^{Q}\left(S_{M;max\;}\times \;W_{M;i\;}\right)}\;\times \text{1}\text{0}\text{0}$$ (5)

Where:

- %HI_M_ is the calculated percentage of Motor Health Index.
- $S_{M;i}$ – is the score of an individual criterion i-th;
- $S_{M;max}$ – is the maximum score for each criterion;
- WCF; C is the weight for an individual criterion i-th;
- Q – is the number of criteria.

### Conditional factor (CF) calculation and parameters

Based on previous studies, expert elections, and the sensitivity and decision impact, the HI calculation for HV Motors only depends on the field testing and inspection or maintenance data, also referred to as visible aging parameters. The visible ageing is the measurable maintenance data, such as dielectric strength of motor winding insulation, mechanical and electrical parameters [31]. Thus, the CF concept used in [5] for GIS health index assessment is proposed to be applied to evaluate the condition of the HV Motor. The CF considers invisible ageing factors such as overall age, overall physical condition, number of mechanical operations, number of fault interruptions, ratio of load current to rated, spare part availability, personnel expertise level for repairing, and Original Equipment Manufacturer OEM support [32].

However, based on industrial best practices and considering easy data availability, only relevant parameters that carry much added value for engineering decisions are being considered in this HV Motor health index proposal. Table 7 below shows the proposed criteria for CF calculation and the details of the score. Aspects proposed to be considered in this study are years in operation, overall physical condition, criticality of motor for plant production, number of breakdowns in the past 10 years, spare availability, and environment of installation. Each aspect is assigned its own weightage based on previous studies and relevant judgment on real case scenarios [33].

**Table 7:** Proposed criteria for conditional Factor CF calculation.

No.	Operating Conditions Factors (CF)	Weightage (%)
1	Years in Operation (YO)	30%
2	Overall physical condition (OC)	10%
3	Criticality of the Motor for the process (CM)	10%
4	Number of Major breakdowns in the past 10 years (NB)	20%
5	Spare part availability (SSA)	10%
6	Environment of Installation (EI)	20%

In this regard, the CF is treated as a separated filed testing. However, there is an overlapping to the diagnostic testing methods. For example, an exposure to the harsh insulation environment can accelerate the insulation degradation, which can be reflected in the measurement on the insulation resistance (IR), Tan Delta (TD), and Partial Discharge (PD). Similarly, the number of historical breakdowns can also degrade the mechanical condition and observed during the diagnostic testing.

A survey conducted in [34] shows that insulation deterioration began to appear gradually in motors that are 6 years or older. The increase was evident from 11 to 15 years, and it rapidly increased for motors 16–20 years or older. The phenomenon seems to gradually decrease after 21 years, was judged to be due to the low failure rate as the motor insulation might have returned to its initial state by performing motor rewinding works. It was confirmed that motors aged 11–25 years have the significantly highest failure rate. Another study in [35] shows highest motor age has the highest failure rate. Thus, the proposed age factor shall be given higher weightage, and the lowest score shall be considered for motors more than 20 years in operation.

Based on studies done for transformers, circuit breakers, and GIS [36], herewith proposed some physical conditions are proposed that indirectly affect the condition of the motor based on actual findings at the site in industry. The condition of the motor frame/housing, cooling fan, fan cowl, air cooling system, and seals are physical conditions that seem to significantly contribute to the overall physical condition. Table 8 describes how to examine the physical condition of the motor for this study. The data shall be taken within 3–6 months to ensure comprehensive H.I calculated. Based on the physical condition of each category, the overall physical condition of the motor is proposed to be 10%.

**Table 8:** Description to examine the physical condition of the motor.

Motor components	Condition Description		
	A	B	C
Motor Housing	The motor body is in good condition.	Minor rust or physical damage on the body is repairable. Repainting required.	Significant Rust or physical damage to the motor body & beyond repair.
Cooling Fan	Cooling fan in perfect condition.	Dirty or minor damage. Repairable.	The fan is damaged, rubbing with the fan cover, or beyond repair.
Fan Cowl unit	Fan cowl unit in good condition.	Significant damage or rust on the cowl unit is repairable.	Significant damage or rust on the cowl unit & beyond repair.
Air cooling unit (CACA cooler)	Cooling tubes and the overall air-cooling unit are in good condition & functioning properly.	Blockages of cooling tubes by dirt. No or minor rust & physical damage observed. Minor servicing required.	Cooling tubes are rusty and damaged. Possible water ingress to the stator compartment. Beyond repair or major repair required.
Seals & gasket	Motor seals and gaskets are in good condition.	Minor physical damage & repairable.	Major physical damage & beyond repair or major repair required.

Critical classifications help prioritize maintenance actions based on the motor’s importance to the production process and potential consequences [37]. Each equipment shall be assessed based on the probability and consequence of its most credible failure. Equipment failure Consequences shall be assessed according to People, Environment, Assets, and Reputation (P, E, A & R). Credible failure scenario, existing counter measure, and impact of the failure are stated. The probability class of the credible failure scenario shall be identified. Criticality 1, Criticality 2, and Criticality 3. They are classified based on the highest risk ranking. Thus, based on [1], PTS and financial loss due to equipment failure, the proposed weightage and scoring description for motor criticality, and the criteria for the criticalities are provided to be 10%.

Based on studies in [1] and [6], the number of failures reported through maintenance work orders affects an equipment’s lifespan. As such, this factor is proposed to be considered as one of the conditional factors too. Breakdowns that shall be considered are Electrical faults that may affect the winding insulation, such as tripping on overload and short circuits. Besides, mechanical defects such as bearing failures and damage to the motor body should be considered. Data shall be extracted from valid tracking, such as the SAP System or equipment reliability history record. Proposed weightage and scoring description for the number of breakdowns is 10%.

Spare part strategy shall be established based on equipment criticality; however, availability of them in a healthy condition and sufficient quantity are usually overlooked, resulting in higher maintenance downtime to repair in case failure occurs in the HV motor during plant operation. Besides, this factor has not been considered in any HV motor health index study so far [38]. Therefore, this study would like to propose weightage and scoring for spare part availability. The data shall be considered critical spare or a whole healthy unit of motor. Critical spare parts are defined as parts that are crucial for the smooth operation of the motor and are prone to wear and tear, such as the bearing and cooling fan. Proposed at least 1 set of bearing, consisting of DE & NDE side, and one piece of cooling fan shall be available in good condition, stored securely with proper preservation to meet scoring 3. Complete new or used-serviced spare unit availability, subject to identical spare, without missing parts, and good health level. Unit shall be stored in proper shaded storage, with appropriate preservation for winding (ensure space heater is energized condition). Electrical health check data on the spare unit within 6 months shall be considered for this study. Healthy condition criteria for score 5 are met if all these are fulfilled. Therefore, the weightage and scoring for spare part availability is 10%

Data from surveys done in [8] and [23] clearly state environment of the motor installation place plays a role as a degrading factor of HV motors. A Higher failure rate was observed for motors located outdoors compared to indoors. Components that usually fail outdoors are bearings and stator windings. Study in [23] concludes that deterioration of high-voltage motors is accelerated when the installation environment is in a seawater area or a dusty area. It was analyzed that motors installed in locations close to seawater have 4–7 times higher failure rate. Motors installed in general environments were analyzed to be relatively low because they are not exposed to the contaminated environment. With that, the weightage and scoring description for the environment of installation is 20%.

As an overview, Table 9 below summarizes the weightage, scoring, and scoring description for the invisible ageing factors being proposed to manipulate the %OHI value of the HV Motor.

**Table 9:** Summary of the weightage, scoring and scoring description.

No.	Operating Conditions	Proposed Weightage%	Scoring Description		
			1	3	5
1	Years in Operation	30	>20 years	10–20 years	< 10 yrs
2	Overall physical condition.	10	Poor	Moderate	Good
3	Criticality of the Motor for the process	10	Criticality 1	Criticality 2	Criticality 3
4	Number of Major breakdowns in the past 10 years	20	>10	5 - 10	<5
5	Spare part availability	10	Spare Motor or parts are unavailable.	Only Critical spare parts are available.	Complete Unit available in a healthy condition.
6	Environment of Installation.	20	Corrosive Environment – Chemical/ Dust/ Sea Water-based.	Outdoor	Indoor

The conditional factor is calculated using the score & weight method, and is calculated using equation (6) below.


$$CF=\frac{\sum_{C=1}^{P}\left(S_{CF;C\;}\times \;W_{CF;C\;}\right)}{\sum_{C=1}^{P}\left(S_{CF;max\;}\times \;W_{CF;C\;}\right)}$$ (6)

where;

- CF is the HV Motor conditional factor.
- $S_{CF;C}$ is the score of an individual criterion c-th;
- $S_{CF;max}$ is the maximum score for each criterion;
- $W_{CF;C}$ is the weight for an individual criterion c-th;
- P is the number of criteria.

### HV motor overall health index (OHIM) calculation

Referring to the methodology used in [6] and applying the weight scoring method, the percentage of overall health index (%OHIM) is calculated. This is by multiplying the obtained percentage Motor Health Index (%HIM) by Conditional Factor (CF), as how being applied to calculate the overall health index of GIS. The overall Motor Health Index with CF is proposed as the equation (7) below.


$${\%OHI}_{M\;}={\%HI}_{M\;}\times \;CF$$ (7)

Where;

- %OHI_M_ is the percentage of the Overall Motor Health Index.
- CF is the Conditional Factor.
- %HI_M_ is the calculated percentage of Motor Health Index.

**Fig 3:**
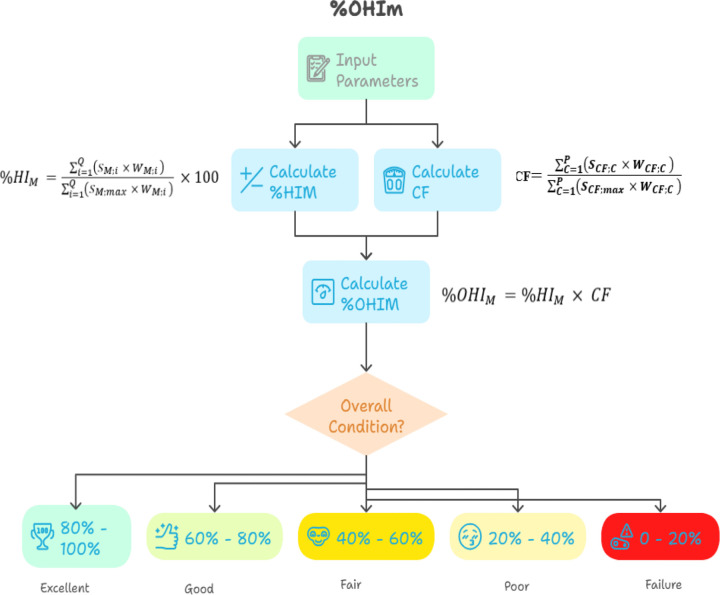
The proposed overall health index OHI_M_ assessment of the HV motors based on electrical testing and conditional factors.

Based on the obtained %OHI_M_ and previous studies [2,3,6,9,10,13] & [23], the life expectancy scale of HV Motor is proposed as Table 10 below. Although the health index is not directly related to life expectancy, but it can give an idea of life expectancy by considering some parameters as constant. To add to the next health index, the criteria are shown in Fig 3 and Table 10. The lifetime estimation of the motor is viewed as a decision-supporting indicator to help engineers to increase attention. The lifetime of the motor shows capital planning rather than forecasting the motor’s end of life.

**Table 10:** Summary overall proposed HV Motor Health Index assessment.

Overall, Health Index (OHI_M_)	Overall condition	Description	Approximate Expected Lifetime	Proposed Mitigation
80% − 100%	Excellent	The motor is in very good condition. Proceed with the existing PPM and periodic testing.	More than 15 years	Proceed with existing PPM and periodic testing.
60% − 80%	Good	The motor is in good condition. Proceed with the existing PPM and increase periodic testing.	More than 10 years	Proceed with the existing PPM and increase periodic testing
40% − 60%	Fair	Motor in fair condition. Increase PPM frequency and routine test.	Up to 10 years	Increase PPM frequency and periodic testing
20% − 40%	Poor	Motor in poor condition. Close monitoring is required on running parameters to avoid failure. Requires service as soon as possible.	Less than 3 years	Close monitoring is required on online parameters to avoid failure. Major servicing required
0 - 20%	Failure	Requires immediate rectification, either replacement or major repair/overhaul of the motor.	At the end of life	Replacement or major repair/overhaul of the motor is required

### Comparison HV motor HI assessment and OHIM

The proposed OHIM is unlike the conventional HV motor health index assessment which rely on the real-time condition monitoring data. The proposed method introduces additional CF to assess the invisible factor of the ageing process under operation conditions. This is mostly not included in the list of measurements directly through any condition testing. These factors include environmental issues, years of operation, spare parts, history of the motor, and critical condition. These additional advantages compared to conventional methods will provide a comprehensive, meaningful health assessment of HV motors. In Table 11, there is a comparison of the existing approaches on HV motor health assessment, and the proposed OHIM method.

**Table 11:** Comparison of existing HV motor HI assessment approaches and OHIM.

Comparison aspect	HI approach/ method (IR, PI, TD, PD)	HI online/data-driven	Proposed OHIM
Data source	Offline test	Real-time data from a sensor	Offline + operational and maintenance data
Considering the CF factors (Ageing, environment, spare parts)	Not considered	Indirect considered	Considered via conditional factor (CF)
Depending on sensors	No	Yes	No sensors required
Applicability to the latest motors	Moderate	Limited	Suitable for all legacy motors
Considering the history of motors	Not considered	Rarely considered	Incorporated
Alignment with standards (IEC, IEEE, CIGRE)	Partial	Often used	Directedly aligned
Interpret the output results	Moderate (depends on expertise)	Model dependent	Decision-oriented OHIM (%)
Supporting maintenance and decision-making	Limited	Fault detection	Do not depend on decision, but prioritize maintenance, spare parts, and replacement.
Suitable for oil& gas industry HV motors	Moderate	Limited by infrastructure	Validated using a real oil/gas industrial

### Experimental studies

There are three experimental case studies to demonstrate the proposed OHI_M_ methodology. The first case study focused on HV Motor GM-151B at 6kV, which drives Boiler Feed Water Pump G-151B, the Pump. The second case study is on HV Motor CM-01004 rated at 6kV, which is used to run C-1004, Nitrogen Off Gas Compressor, and the third is HV Motor GM-06501B 6kV, which runs pump G-06501B, Sea Water Pump.

### Case Study 1: HV motor GM-151B

**Fig 4:**
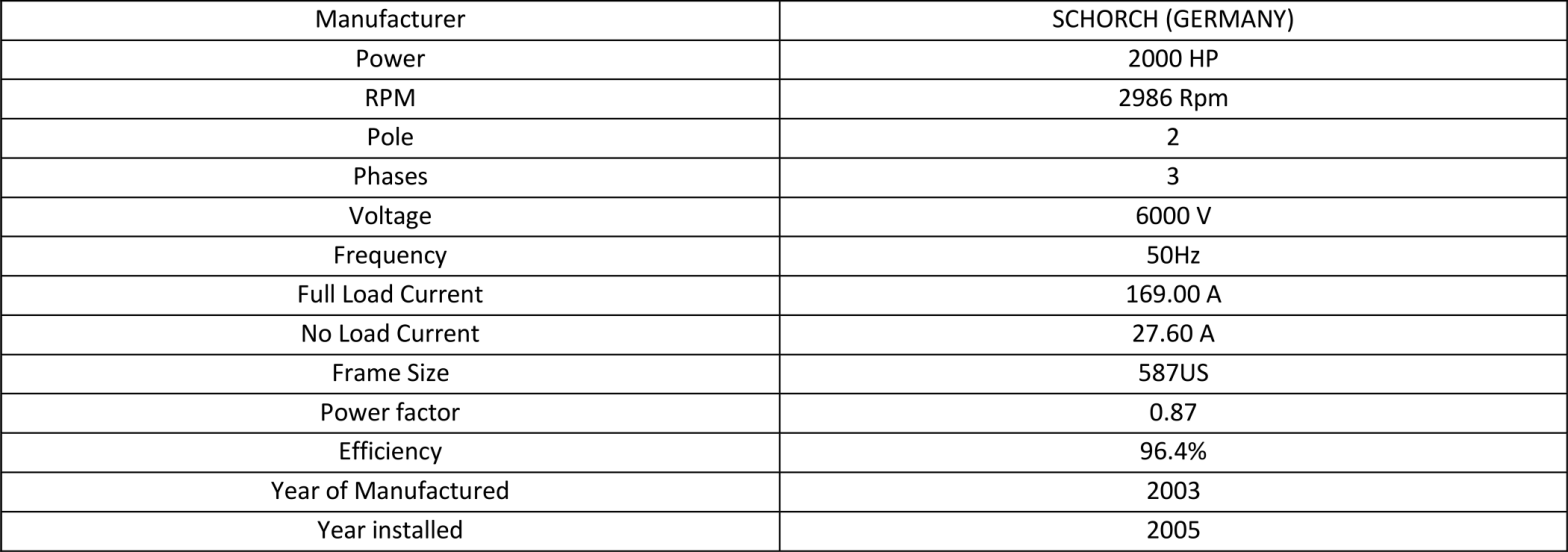
GM-151B is an HV Motor used to run pump G-151B, the Boiler Feed Water name Plate Details.

GM-151B is an HV Motor used to run pump G-151B, the Boiler Feed Water Pump processing under the Steam Reforming unit shown in Fig 4. An operating philosophy for this motor is required during plant start-up, as it is unable to start the plant without this motor. It’ll be running until the steam system is available and will swing over to pump GM-151A, driven by a mini steam turbine. In case of low pressure or sudden issue with GM-151A, this pump will kick in to build up pressure immediately. Based on process requirements, loss of boiler feed water will trip the front-end process, subsequently tripping the plant system, leading to millions $ lost. Thus, it is always required to be healthy and unable to be serviced during plant operation. Due to the nature of the operating philosophy, this Motor is categorized as Criticality 1 under Equipment Criticality Analysis (ECA) [39].

The motor’s maintenance record obtained for Health Index calculation is as tabulated in Table 12. By assigning respective scores for the parameters, we can calculate the health index as in Table 13. The Percentage of HV Motor Health Index (%HI_M_) is calculated by using Equation (5)

**Table 12:** GM-151B Maintenance Record Obtained from Case Study for Health Index Calculations.

No.	Parameters	Result
1	Insulation Resistance (I.R)	2.4 GΩ
2	Polarization Index (P.I)	3.19
3	Tan Delta (T.D) at 0.2Un	1.2739
4	Partial Discharge (P.D)	3000
5	Vibration Analysis (VA)	Good

**Table 13:** Results converted into Health Index scoring table for motor GM-151B.

Motor Health Index (%HI_M_)										
No	Type of Test	Weightage (%)	Scoring Range & Description			Result	Ideal Score	Ideal Result	Actual Score	Actual Result
			1	3	5					
1	Insulation Resistance (I.R)	30	< 2(kV + 1) MΩ	2(kV + 1) MΩ – 1GΩ	>1 GO	2.4 GΩ	5	1.5	5	1.5
2	Polarization Index (PI)	20	<2	2-3	>3	3.19	5	1	5	1
3	Tan Delta	20	>10%	10% 2%	<2%	3.27%	5	1	3	0.6
4	Partial Discharge	20	>20000	20000−5000-	<5000	3000	5	1	5	1
5	Vibration Analysis	10	Danger	Alert	Good	Good	5	0.5	5	0.5
Total							25	5	23	4.6
Motor Health Index (%HI_M_) $=\frac{\sum_{i=1}^{Q}\left(S_{M;i\;}\times \;W_{M;i\;}\right)}{\sum_{i=1}^{Q}\left(S_{M;max\;}\times \;W_{M;i\;}\right)}\;\times 100$							**92%**			

The motor’s maintenance record obtained for Conditional Factor calculation is as per tabulated in Table 14. By assigning respective scoring for the parameter’s result, we can calculate the conditional factor as Table 15.

**Table 14:** GM-151B Maintenance Record Obtained for Conditional Factor Calculations.

No	Parameters	Result
1	Years in Operation (YP)	21 years
2	Overall physical condition (OPC)	Good
3	Criticality of the Motor for the process (CM	Criticality 1
4	Number of Major breakdowns in the past 10 years (MB)	4 breakdowns
5	Spare part availability (SSA)	Only 1 set of DE & NDE bearing available
6	Environment of Installation (EI)	Outdoor

**Table 15:** Results Converted into Conditional Factor Scoring Table for GM-151B.

Conditional Factor (C.F)										
No	Parameters	Weightage %	Scoring Range & Description			Result	Ideal Score	Ideal Result	Actual Score	Actual Result
			1	3	5					
1	Years in Operation	30	>20 years	10 - 20 years	< 10 years	21 years	5	1.5	1	0.3
2	Overall physical condition.	10	Poor	Moderate	Good	Good	5	0.5	5	0.5
3	Criticality of the Motor for the process.	10	Criticality 1	Criticality 2	Criticality 3	Criticality 1	5	0.5	1	0.1
4	Number of Major breakdowns in the past 10 years	20	>10	5 - 10	<5	4 breakdowns	5	1	5	1
5	Spare Part Availability	10	Spare Motor or parts are unavailable.	Only Critical spare parts are available.	Complete Unit available in a healthy condition.	DE & NDE Bearing Available	5	0.5	3	0.3
6	Environment of Installation.	20	Corrosive Environment – Chemical/ Sea Water-based.	Outdoor	Indoor	Outdoor	5	1	3	0.6
Total							30	5	18	2.8
**Conditional Factor, CF** $=\frac{\sum_{C=1}^{P}\left(S_{CF;C\;}\times \;W_{CF;C\;}\right)}{\sum_{C=1}^{P}\left(S_{CF;max\;}\times \;W_{CF;C\;}\right)}$							**0.56**			

The percentage of overall health index (%OHI_M_) is calculated by multiplying the obtained percentage Motor Health Index (%HI_M_) by the Conditional Factor (CF) as shown in Equation (7).


$${\%OHI}_{M\;}={\%HI}_{M\;}\times \;CF\;=\;52\%$$

Based on the proposed Table 10, the Life expectancy scale of HV Motor, and based on the obtained overall GM-151B motor health index of 52%, it can be concluded that the condition falls under fair condition, where the approximate expected lifetime of the motor is predicted up to 10 years. It is proposed to increase preventive maintenance frequency and periodic testing. In this regard, the HI values under testing is 92%, but due to the lower condition of CF due to years of operation more than 20 years the overall condition would be affect the du to the influence of aging, harsh insulation, and breakdown history. Therefore, the OHIM is scouring 52%, which recommends increasing the preventive maintenance.

### Case Study 2: HV Motor CM-01004

**Fig 5:**
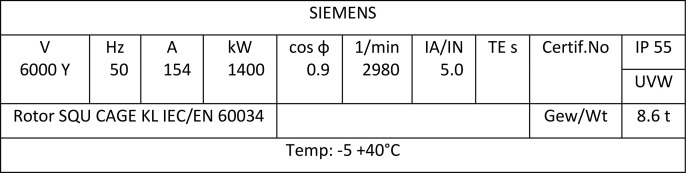
CM-01004 is an HV Motor used to run C-1004, Nitrogen Off Gas Compressor name Plate Details.

CM-01004 is an HV Motor used to run C-1004, Gas Compressor, shown in Fig 5. It is a horizontal motor, attached to the compressor shaft via a sleeve bearing. Operating philosophy for this motor. It is required during the plant start-up and shutdown. It is possible to start the plant without this motor, but it’ll take extra days to have a production outcome. The mode of operation is categorized as run when required, so the running hours for this motor are significantly low. It is also located inside a closed cubicle. Hence, this Motor is categorized as Criticality 1 under Equipment Criticality Analysis (ECA) studies [40].

The motor’s maintenance record obtained for Health Index calculation is tabulated in Table 16. By assigning respective scores for the parameters, we can calculate the health index as in Table 17. The Percentage of HV Motor Health Index (%HIM) is calculated by using Equation (5).

**Table 16:** CM-01004 maintenance record obtained from case study for health index Calculations.

No.	Parameters	Result
1	Insulation Resistance (I.R)	3.7 GΩ
2	Polarization Index (P.I)	4.08
3	Tan Delta (T.D) at 0.2Un	0.7725%
4	Partial Discharge (P.D)	4000
5	Vibration Analysis (VA)	Good

**Table 17:** Results converted into Health Index scoring table for motor CM-01004.

Motor Health Index (%HI_M_)										
No	Type of Test	Weightage (%)	Scoring Range & Description			Result	Ideal Score	Ideal Result	Actual Score	Actual Result
			1	3	5					
1	Insulation Resistance (I.R)	30	< 2(kV + 1) MΩ	2(kV + 1) MΩ – 1GΩ	>1 GO	3.7 GΩ	5	1.5	5	1.5
2	Polarization Index (PI)	20	<2	2-3	>3	4.08	5	1	5	1
3	Tan Delta	20	>10%	10% 2%	<2%	0.77%	5	1	5	1
4	Partial Discharge	20	>20000	20000−5000	<5000	1000	5	1	5	1
5	Vibration Analysis	10	Danger	Alert	Good	Good	5	0.5	5	0.5
Total							25	5	25	5
The Motor Health Index (%HI_M_) $=\frac{\sum_{i=1}^{Q}\left(S_{M;i\;}\times \;W_{M;i\;}\right)}{\sum_{i=1}^{Q}\left(S_{M;max\;}\times \;W_{M;i\;}\right)}\;\times 100$							**100%**			

The CM-01004 Motor’s maintenance record obtained for Conditional Factor calculation is as per tabulated in Table 18. By assigning respective scoring for the parameter’s result, we can calculate the conditional factor for CM-01004 as Table 19.

**Table 18:** GM-151B maintenance record obtained for conditional factor calculations.

No	Parameters	Result
1	Years in Operation (YP)	5 years
2	Overall physical condition (OPC)	Good
3	Criticality of the Motor for the process (CM	Criticality 2
4	Number of Major breakdowns in the past 10 years (MB)	No major breakdowns
5	Spare part availability (SSA)	1 set of DE & NDE bearing available.
6	Environment of Installation (EI)	Indoor/ Enclosed

**Table 19:** Results converted into conditional factor scoring table for GM-151B.

Conditional Factor (C.F)										
No	Parameters	Weightage %	Scoring Range & Description			Result	Ideal Score	Ideal Result	Actual Score	Actual Result
			1	3	5					
1	Years in Operation	30	>20 years	10 - 20 years	< 10 years	5 years	5	1.5	5	1.5
2	Overall physical condition.	10	Poor	Moderate	Good	Good	5	0.5	5	0.5
3	Criticality of the Motor for the process.	10	Criticality 1	Criticality 2	Criticality 3	Criticality 2	5	0.5	3	0.3
4	Number of Major breakdowns in the past 10 years	20	>10	5 - 10	<5	0 breakdowns	5	1	5	1
5	Spare Part Availability	10	Spare Motor or parts are unavailable.	Only Critical spare parts are available.	Complete Unit available in a healthy condition.	DE & NDE Bearing Available	5	0.5	3	0.3
6	Environment of Installation.	20	Corrosive Environment – Chemical/ Sea Water-based.	Outdoor	Indoor	Indoor/ Enclosed	5	1	5	1
Total							30	5	26	4.6
**Conditional Factor, CF** $=\frac{\sum_{C=1}^{P}\left(S_{CF;C\;}\times \;W_{CF;C\;}\right)}{\sum_{C=1}^{P}\left(S_{CF;max\;}\times \;W_{CF;C\;}\right)}$							**0.92**			

The percentage of overall health index (%OHIM) is calculated by multiplying the obtained percentage Motor Health Index (%HIM) by the Conditional Factor (CF) as shown in Equation (7).


$${\%OHI}_{M\;}={\%HI}_{M\;}\times \;CF\;=\;92\%$$

By referring to Table 10, the life expectancy scale of HV Motor, and based on the calculated overall motor health index of 92% falls under the excellent category, where the approximate expected lifetime of the motor is predicted to be more than 15 years. It is proposed to proceed with the existing PPM and periodic testing, such as yearly CBM and bearing inspections during turnarounds. The motor CM-01004 maintains both a high HI (100%) and OHIM (92%), indicating that CF does not unnecessarily penalize assets operating in favorable conditions. This confirms that CF integration improves decision selectivity. The motor is less than 10 years and other CF are in good condition.

### Case Study 3: HV Motor GM-06501B

**Fig 6:**
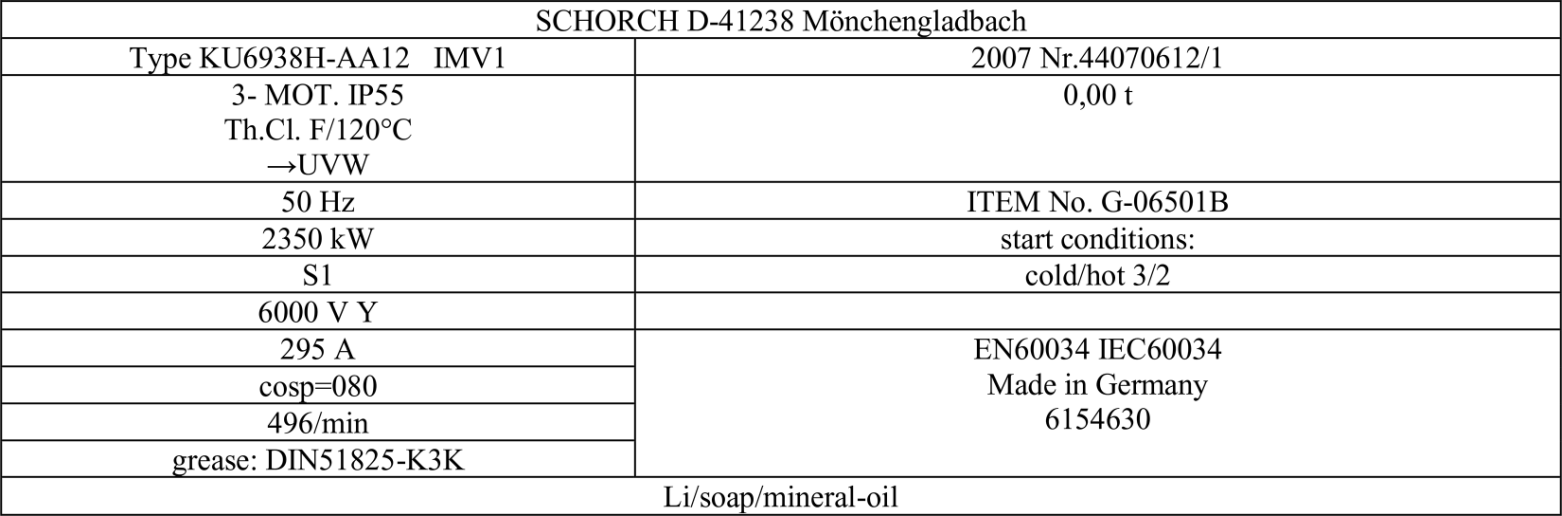
GM-0651B is an HV Motor used to run pump G-06501B, Sea Water Pump (a) Site Picture of HV Motor (b) Name Plate Details.

GM-0651B is an HV Motor used to run pump G-06501B, Sea Water Pump, as shown in Fig 6. This pump is a vertical pump, located near the seaside, commissioned in the year 2008, used to pump the seawater from the sea to the plant under the seawater cooling water system. Sea water is used in cooling mechanical and process equipment such as heat exchangers. Besides, it uses water as a cooling medium for the motor itself [41,42]. As for operating philosophy, there are three Sea Water Pumps, which are GM-06501A, GM-06501B, and GM-06501C, designed for the continuous operation of two pumps at a time with a monthly swing schedule. Due to the availability of a standby pump and does not cost a major impact in case of failure, this Motor is categorized as Criticality 3 under Equipment Criticality Analysis (ECA) studies.

Health Index calculation is in Table 20. By assigning respective scores for the parameters, we can calculate the health index as in Table 21. The Percentage of HV Motor Health Index (%HI_M_) is calculated by using Equation (5).

**Table 20:** CM-01004 maintenance record obtained from case study for health index calculations.

No.	Parameters	Result
1	Insulation Resistance (I.R)	598 MΩ
2	Polarization Index (P.I)	4.10
3	Tan Delta (T.D) at 0.2Un	6.7476%
4	Partial Discharge (P.D)	4000
5	Vibration Analysis (VA)	Good

**Table 21:** Results converted into Health Index scoring table for motor CM-01004.

Motor Health Index (%HI_M_)										
No	Type of Test	Weightage (%)	Scoring Range & Description			Result	Ideal Score	Ideal Result	Actual Score	Actual Result
			1	3	5					
1	Insulation Resistance (I.R)	30	< 2(kV + 1) MΩ	2(kV + 1) MΩ – 1GΩ	>1 GO	598 M Ω	5	1.5	3	0.9
2	Polarization Index (PI)	20	<2	2-3	>3	4.10	5	1	5	1
3	Tan Delta	20	>10%	10% 2%	<2%	6.75%	5	1	3	0.6
4	Partial Discharge	20	>20000	20000−5000	<5000	4000	5	1	5	1
5	Vibration Analysis	10	Danger	Alert	Good	Good	5	0.5	5	0.5
Total							25	5	21	4
The Motor Health Index (%HI_M_) $=\frac{\sum_{i=1}^{Q}\left(S_{M;i\;}\times \;W_{M;i\;}\right)}{\sum_{i=1}^{Q}\left(S_{M;max\;}\times \;W_{M;i\;}\right)}\;\times 100$							**80%**			

The CM-01004 Motor’s maintenance record obtained for Conditional Factor calculation are as per tabulated in Table 22. By assigning respective scoring for the parameter’s result, we can calculate the conditional factor for CM-01004 as Table 23.

**Table 22:** GM-151B maintenance record obtained for conditional factor calculations.

No	Parameters	Result
1	Years in Operation (YP)	16 years
2	Overall physical condition (OPC)	Moderate
3	Criticality of the Motor for the process (CM)	Criticality 3
4	Number of Major breakdowns in the past 10 years (MB)	8 breakdowns
5	Spare part availability (SSA)	Only 1 set of DE & NDE bearing available.
6	Environment of Installation (EI)	Corrosive Environment – Sea Water

**Table 23:** Results converted into conditional factor scoring table for GM-151B.

Conditional Factor (C.F)										
No	Parameters	Weightage %	Scoring Range & Description			Result	Ideal Score	Ideal Result	Actual Score	Actual Result
			1	3	5					
1	Years in Operation	30	>20 years	10 - 20 years	< 10 years	16 years	5	1.5	3	0.9
2	Overall physical condition.	10	Poor	Moderate	Good	Moderate	5	0.5	3	0.3
3	Criticality of the Motor for the process.	10	Criticality 1	Criticality 2	Criticality 3	Criticality 3	5	0.5	5	0.5
4	Number of Major breakdowns in the past 10 years	20	>10	5 - 10	<5	8 breakdowns	5	1	3	0.6
5	Spare Part Availability	10	Spare Motor or parts are unavailable.	Only Critical spare parts are available.	Complete Unit available in a healthy condition.	Only 1 DE & NDE Bearing Available	5	0.5	3	0.3
6	Environment of Installation.	20	Corrosive Environment – Chemical/ Sea Water-based.	Outdoor	Indoor	Sea Water	5	1	1	0.2
Total							30	5	18	2.8
Conditional Factor, CF$=\frac{\sum_{C=1}^{P}\left(S_{CF;C\;}\times \;W_{CF;C\;}\right)}{\sum_{C=1}^{P}\left(S_{CF;max\;}\times \;W_{CF;C\;}\right)}$ = 2.8/5.0							0.56			

The percentage of overall health index (%OHI_M_) is calculated by multiplying the obtained percentage Motor Health Index (%HI_M_) by the Conditional Factor (CF) as shown in Equation (7).


$${\%OHI}_{M\;}={\%HI}_{M\;}\times \;CF\;=\;45\%$$

By referring to Table 10, the life expectancy scale of HV Motor, and based on the obtained overall motor health index of 45%, it can be concluded that the condition falls under fair condition, where the approximate expected lifetime of the motor is predicted up to 10 years. It is proposed to increase preventive maintenance frequency and periodic testing. The CF reflects the influence of the advanced age of the motor at 16 years, harsh installation environment, breakdown history, and limited spare availability. This quantitative reduction directly alters the recommended actions, prompting increased inspection frequency.

### Analysis of the (OHI_M_) Patterns

**Fig 7:**
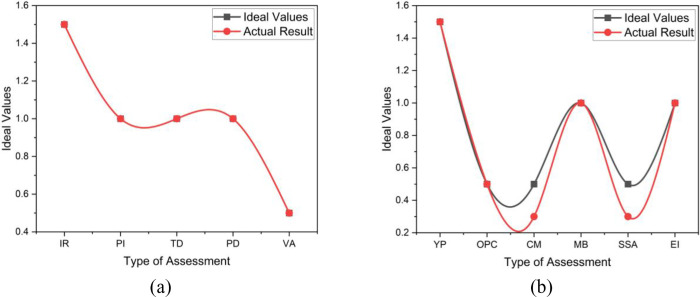
The OHI_M_ Results patterns of HV Motor GM-151B (a) HI tests assessment (b) Conditional factor CF calculation.

Based on the overall health index calculation of motor GM-151B, we can see that even though the health index of the motor itself is 92%, it falls under the excellent category as Fig 7a indicates the variation between the ideal and actual results on the Insulation resistance (IR) and the Tan Delta (TD) tests and the other tests show identical to ideal results such as the Polarization Index (PI), Partial Discharge (PD) and the Vibration Analysis. But, after considering the CF of 0.56, the Overall Health Index (OHI) actually further falls to 55%, under the fair category. The variation between the ideal and actual results of CF parameters is shown in Fig 7b. This HV motor only has the Overall physical condition (OPC) and Number of Major breakdowns in the past 10 years (MB) identical to ideal results. Although it is a C1 equipment, the electrical health of the motor looks convincing might be due to not continuously operating, frequent PPM compared to the C3 motor, and being located in a normal outdoor environment. However, due to higher years in operation and only critical spare parts are available for this motor, it is highly recommended to procure a complete new set as a worthwhile capital investment. Besides, constructing a shade for the motor might help improve the conditional factor and indirectly improve the motor’s overall health index.

**Fig 8:**
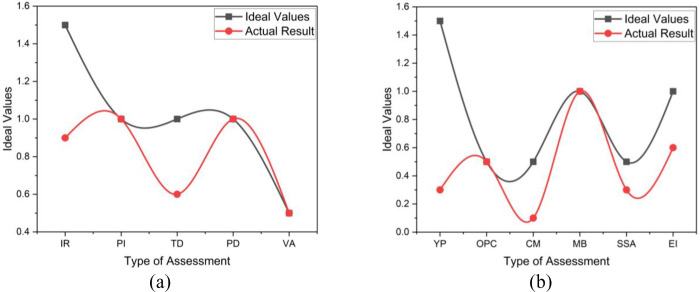
The OHI_M_ Results patterns of HV Motor CM-01004 (a) HI test assessment (b) Conditional factor CF calculation.

For HV Motor CM-01004, it can be concluded that both the motor and the overall health index are at an excellent level. Fig 8a shows the identical patterns between the ideal and actual results. The electrical health of the motor looks very convincing due to not continuously operating, frequent PPM conducted, compared to the C3 motor, and located in an indoor or enclosed environment. As the motor’s scores are all rated 5, a 100% healthiness is achieved. Other conditional factors do not affect much on the overall H.I_M_ as the years in operation are low, and the motor is located in a non-contaminated cubicle. The variation in the results shown in Fig 8b is due to the HV motor criticality of the HV motor (CM), and the availability of spare (SSA). Thus, the need for a complete spare unit for this motor can be put on hold as the motor condition is excellent and no complete spare is required cost-effective.

**Fig 9:**
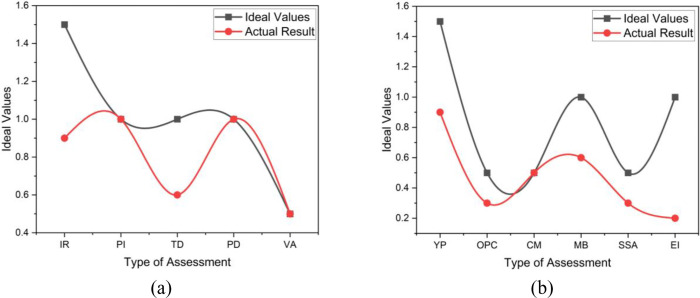
The OHI_M_ Results patterns of HV Motor GM-06501B (a) HI test assessment (b) Conditional factor CF calculation.

Based on the obtained motor health index for GM-06501B, despite the IR and TD values being slightly low, the motor health index was 80%, which falls under the excellent category, as actual results shown in Fig 9a. But after considering the CF of 0.56, the Overall Health Index actually further falls to 45%, under the fair category, as Fig 9b shows the difference in ideal and actual results. It can be analyzed that the major contributor to the lower CF value on this motor is contributed by several breakdowns and the environment of the motor, which is located near the seawater environment. Referring to the breakdown record, this motor has been overhauled a few times due to bearing failure, which indirectly relates to the study in [23], which stated that the motor installed in locations close to seawater has a 4–7 times higher failure rate. Besides, it is not a critical motor for the process; constructing an enclosure for the motor might help improve the conditional factor and indirectly improve the motor’s overall health index from poor to fair or good category.

Overall, even though the motors are in excellent health condition, after considering conditional factors, most of the motors’ comprehensive health condition seems to degrade one stage at a time. Thus, it proves that the conditional factor does play a role in determining the Overall Health Index of an asset, which may indirectly affect it in the long run.

## Conclusion

Based on the proposed health index methodology, by having a comprehensive health index, Engineers can make clear quantitative decisions on what to perform on that HV Motor at a particular point in time, either to perform minor service, major inspection, major service, or purchase and replace a new unit. It also helps strengthen the oversight of critical assets, which may lead to failure, consequently, plant upset. It’ll also be a dynamic tool for decision-making since the input data can be manipulated based on the current time. It’ll show the current condition of the motor and what could be the condition of the motor and its preventive action required for the next 5 years, provided similar operating method, operating condition, and maintenance strategy are applied. By using the proposed (OHI_M_) health index, maintenance can be scheduled proactively rather than reactively. This helps in preventing unexpected failures and reducing downtime. Besides, it, also helps in predicting the remaining life expectancy of the motor and enables data-driven decision-making. In a nutshell, relevant parameters to be considered for HV Motor Health Index and its Conditional Factor have been discussed, formulation for Comprehensive Motor Health Index using weight & scoring method has been developed, and the formula has been applied on a real case scenario for several HV motors. It provides data support to the overall objective of this paper. Three oil & gas HV motors studied and GM-151B, GM-06501B, and CM-01004, the first two are in fair condition and proposed to increase PPM frequency and routine test. Also expected the lifetime to be 10 years. The third CM-01004 is in excellent condition, and the motor is in very good condition. The proposed recommendation is to proceed with the existing PPM and periodic testing. Also expected lifetime can be extended to 15 years. This approach will broaden horizons on the health and safety of HV equipment and reduce the unplanned downtime and maintenance costs, and ensure the high performance of HV motors in industry applications.
